# Effects of work environments on satisfaction of nurses working for integrated care system in South Korea: a multisite cross-sectional investigation

**DOI:** 10.1186/s12912-024-02075-9

**Published:** 2024-07-08

**Authors:** Jinhyun Kim, Eunhee Lee, Hyunjeong Kwon, Sunmi Lee, Hayoung Choi

**Affiliations:** 1 College of Nursing, Seoul National University, Seoul, Korea; 2 College of Nursing, Sungshin Women’s University, Seoul, Korea; 3 College of Nursing, Keimyung University, Daegu, Korea

**Keywords:** Nurse, Satisfaction, Work environment, Workload, Delegation

## Abstract

**Background:**

Nurses’ satisfaction has an impact on organizational and patient outcomes. Integrated care system in South Korea was established in 2015 to improve care quality and decrease caregiving burden. Since then, nurses’ satisfaction has increased due to an increase in nursing staffing. However, besides nurse staffing, various work environments still affect nurse satisfaction.

**Methods:**

Individual online surveys were conducted with participants to determine their personal characteristics, work environments, and hospital characteristics. We used mixed-effects linear regression equation contained both fixed and random effects.

**Results:**

This study included 2,913 nurses from 119 hospitals. Their average job satisfaction was less than 6 points out of 10 points. Age, shift type, perceived workload, and delegation criteria were significant factors influencing nurses’ satisfaction. There was no significant factor among hospital characteristics. The satisfaction level of nurses was high for no-night rotating shift, low perceived workload, and clear delegation criteria.

**Conclusions:**

Nurses’ satisfaction is affected by several work environmental factors. Low nurse satisfaction has a substantial impact on both patients and nurses. Therefore, nurse managers and hospitals should determine factors influencing their satisfaction and develop strategies to improve their satisfaction.

## Background

Job satisfaction has a direct impact on organizational outcomes. It also has a potential impact on the quality and safety of patient care [1]. Job satisfaction has already been revealed to be a factor that can directly influence nurses’ turnover intention among several organizational outcomes such as turnover, sickness absence, and job performance [1–3]. Low job satisfaction is directly related to the actual turnover of nurses [2], whereas a high job satisfaction contributes to better work performance and organizational outcomes, improved work productivity, and reduced absenteeism [1, 4]. Nurses’ job satisfaction also plays an important role in organization outcomes. A high job satisfaction can lead to better patient outcomes and improved patients’ satisfaction with the quality of nursing care [1]. Therefore, hospitals need to proactively manage nurses’ job satisfaction to improve organization outcomes and patient outcomes.

Nurses’ job satisfaction is known to be affected by various factors. Most studies have reported that organizational factors affecting workload such as nurse staffing, work shift, and overtime with a major impact on nurses’ satisfaction [5–7]. Better nurse staffing has already been revealed to be a factor directly influencing several nurse outcomes, including job satisfaction, burnout, and turnover [5, 8, 9]. Besides nurse staffing, work shift also influences nurses’ satisfaction. Nurses who work 12 h or more are likely to be dissatisfied with their job than nurses who work 8 h or less [10]. In addition, job satisfaction of nurses who work rotation shifts including night shift is low due to poor sleep quality and health [11, 12]. Hence, workload determined by staffing and work shifts can directly influence nurses’ job satisfaction. In addition to the work environment, socio-cultural environments such as manager’s leadership, organizational commitment, effort, and reward style have also been identified as factors influencing job satisfaction [1].

The Korean government started to implement an integrated care system to provide care service with a drastically increased nursing staff in 2013 and expanded this system nationwide [13]. This care system provides not only basic care, but also highly technical care. Such care is delivered by registered nurses (RN), nurses’ aides (NA), and other staff. Without caregiver concerns, patients can be admitted to an integrated care unit regardless of the medical department. Nurse staffing standards in integrated care units are different depending on hospital types and characteristics [14]. Nurse staffing standards in an integrated care unit are the highest in tertiary hospital, where there are many patients with high nursing needs, followed by those in general hospitals and semi-hospitals. An RN takes care of 5–7 patients in a tertiary hospital, 7–12 patients in a general hospital, and 10–16 patients in a semi-hospital according to staffing standards [14]. In addition, an NA takes care of 30–40 patients in a tertiary hospital and 25–40 patients in a general hospital or semi-hospital [13]. Nursing staff in Korea consists of RN and NA, not licensed practical nurses (LPNs). Thus, RNs handle all skilled nursing such as assessment, medication, and intervention and NAs handle personal care, including assistance with ADL.

Korea’s integrated care system, in which the level of nursing staffing is increased, has substantially improved nurse performance, including nurse satisfaction [6, 14]. Nurses’ satisfaction in integrated care units with better nurse staffing is higher than that in general units. However, even within integrated care units, as the level of nurse staffing is different with diverse working methods, and working environments, these various work environments can affect nurse satisfaction. Although most studies have identified the commonality of why nurses may intend to leave positions due to lack of job satisfaction, there is still not a clear picture of how different variables can predict job satisfaction. Moreover, several factors have complex effects on nurses’ job satisfaction. However, previous studies did not consider different factors simultaneously. Thus, the aim of this study was to determine complex effects of personal, work environment, and organizational factors on nurses’ job satisfaction.

## Methods

### Participants and survey

This study aimed to determine effects of personal factors, work environment factors, and hospital factors on job satisfaction of nurses who were working in integrated care units in South Korea. Nurses who were working in an integrated care unit from all hospitals (504 hospitals) were invited to participate in this study, considering various factors related to their work environment. The purpose of the survey was explained to all hospitals having integrated care units. An online survey link was distributed only to those who voluntarily agreed to participate in the survey. In August 2020, an online survey was conducted to collect information on job satisfaction, personal characteristics, working environment, and hospital characteristics. A total of 2,913 nurses from 119 hospitals, including 18 tertiary hospitals, 67 general hospitals, and 34 semi-hospitals, were included in this study (hospital participation rate: 23.6%).

### Variables

We included nurses’ satisfaction and three types of influencing factors (personal, work environment, and hospital factors). Nurses’ satisfaction was measured with a 10-point Likert scale ranging from strongly dissatisfied (a score of 1) to strongly satisfied (a score of 10). Personal characteristics included age, gender, and work experience in the current department.

The work environment was categorized as shifts, employment type, salary, perceived workload, nursing care delivery, practice guidelines, and deviation from staffing standards. Nursing shifts were classified into five types: rotating three shifts, rotating two shifts, no-night rotating shift, night-fixed shift, and fixed shift. All nursing shifts are 8-hour shifts except for the type of two shifts (12-hour shifts). Most nurses work 40 h per week. Those with night-fixed shifts work 32 h per week. Perceived workload was measured with a 5-point Likert scale ranging from too low (a score of 1) to too high (a score of 5). Nursing care delivery was the method for organizing and delivering nursing care to patients. It was classified into patient allocation and task allocation in this study. Practice guidelines included the scope of practice among nurses, nursing assistants, and support staffs in integrated units. Deviation from staffing standards was measured to determine whether the average number of patients in charge per day met staffing standards. Since staffing standard was the ratio of the average number of nurses per shift to the average number of patients per shift per day, the number of patients in charge of each shift might exceed the staffing standard. We surveyed the average number of patients in charge by shift for the past month. Subsequently, we calculated the daily average number of patients in charge and evaluated whether the staffing standard was met or not.

Hospital characteristics included hospital type, bed size, staffing standards, and participating COVID-19 care. Staffing standards in integrated care units are applied differently by hospital type. They are constant at the unit level. Hospitals can select a nurse-to-patient ratio for RN staffing between 1:5 and 1:7 for a tertiary hospital, between 1:7 and 1:12 for a general hospital, and between 1:10 and 1:16 for a semi-hospital. NA staffing is between 1:30 and 1:40 for a tertiary hospital and betweeen1:25 and 1:40 for a general hospital or a semi-hospital. The nurse-to-patient ratio was transformed to hours per patient day (HPPD) by RN and NA, which was calculated as total hours (24 h) of a day divided by the number of patients under a nurse staff’s charge. As Korea designates hospitals for COVID-19 patients, we investigated whether the hospital was a designated hospital or not.

### Ethical consideration

This study received ethical approval from Seoul National University Institutional Review Board (No. 2008/002–010). Written informed consent for the survey was obtained from all participants.

### Analysis

Personal, work environment, hospital characteristics are reported as mean and standard deviation (SD) for continuous variables and frequencies and percentages for categorical variables. We used a chi-squared test and ANOVA to determine whether nurses’ characteristics differed significantly by hospital type. In case of staffing standards, the nurse-to-patient ratio was reported in descriptive analyses and HPPD translated from the ratio of patients to total nursing personnel including nurses and nurse assistants was used in the regression model. Mixed-effects regression model was used to identify factors affecting nurses’ satisfaction.

In this study, we first targeted hospitals that operated integrated care units. We then conducted a survey for nurses who worked in those integrated care units. Thus, analytic data consisted of two-level data (nurse data and hospital data). Mixed-effects linear regression equation contained both fixed and random effects. In these structures, such as two-level, longitudinal, and panel data, random effects are useful for modeling intra-cluster correlation. In this study, observations in the same hospital were correlated because they shared common hospital-level random effects. Thus, a mixed-effect linear regression equation was appropriate for this study because it could consider both fixed coefficients for predictor variables and random effects for each hospital. To be specific, the random-intercept model, a type of mixed-effects model, was produced to consider clustered and unbalanced structure of the data. The model has the advantage of dealing with correlations of observations within a subject, in this case, quarters within a hospital [15]. Stata SE version 14.2 was used for all statistical analyses.

## Results

### Nurses’ characteristics in integrated care units

Table 1 presents characteristics of 2,913 nurses working in integrated care units in South Korea. Nurses working in general hospitals had the highest percentage (60.9%, 1,775/2,913), followed by those working for tertiary hospitals (25.7%, 748/2,913) and semi-hospitals (13.4%, 380/2,913). The majority (95.9%) of nurses were women. The average of all nurses was 30.0 years. The proportion of nurses who worked within one year was approximately 30%. This percentage was similar regardless of the type of hospital. As most Korean hospitals operate in three shifts (day, evening, and night shifts), three shifts accounted for the most (more than 80%) of nurses included in this study, followed by day or evening shift (7.8%), night shift (3.8%), two shifts (1.2%), and fixed shift (0.8%). Most nurses were employed full-time. The proportion of nurses with a monthly salary of 3 million won or more was the highest for nurses in tertiary hospitals. More than 60% of nurses answered that the workload was high or very high. The proportion of nurses who answered that the workload was high or very high was the highest in general hospitals (73.3%). Most nurses (79.6%) were assigned patients (patient allocation), while task allocation still accounted for 20%. Most (81.8%) nurses responded that the hospital had work guidelines. This result was similar regardless of the type of hospital. Although more than 70% of nurses did not take care of patients exceeding the staffing standard, approximately 30% of nurses took care of patients above the staffing standard except for those working in tertiary hospitals.

**Table 1:** Characteristics of nurses in integrated care unit and their work environment

		Total (*n* = 2913)		Tertiary hospital (*n* = 748)		General hospital (*n* = 1775)		Semi-hospital (*n* = 390)		Chi^2^	*p*-value
**Personal factors**											
Gender	Male	120	(4.1)	9	(1.2)	98	(5.5)	13	(3.3)	53.697	< 0.001
	Female	2,793	(95.9)	739	(98.8)	1,677	(94.5)	377	(96.7)		
Age	Mean ± S.D.	30.0 ± 7.3		29.2 ± 6.6		29.7 ± 7.2		32.7 ± 8.4			
	< 30 years	1,857	(63.7)	502	(67.1)	1,166	(65.7)	189	(48.5)	53.697	< 0.001
	31–39 years	702	(24.1)	179	(23.9)	400	(22.5)	123	(31.5)		
	≥ 40 years	354	(12.2)	67	(9.0)	209	(11.8)	78	(20.0)		
Work experience in current department	< 1 year	898	(30.8)	252	(33.7)	520	(29.3)	126	(32.3)	10.647	0.100
	1–2 years	812	(27.9)	202	(27.0)	514	(29.0)	96	(24.6)		
	2–3 years	432	(14.8)	92	(12.3)	277	(15.6)	63	(16.2)		
	≥ 3 years	771	(26.5)	202	(27.0)	464	(26.1)	105	(26.9)		
**Work environment factors**											
Shift	Rotating three shifts	2,519	(86.5)	707	(94.5)	1,545	(87.0)	267	(68.5)	163.759	< 0.001
	Rotating two shifts	34	(1.2)	10	(1.3)	18	(1.0)	6	(1.5)		
	No-night rotating shift	228	(7.8)	22	(2.9)	129	(7.3)	77	(19.7)		
	Night-fixed shift	110	(3.8)	8	(1.1)	67	(3.8)	35	(9.0)		
	Fixed shift	22	(0.8)	1	(0.1)	16	(0.9)	5	(1.3)		
Employment type	Part-time	67	(2.3)	32	(4.3)	28	(1.6)	7	(1.8)	17.591	< 0.001
	Full-time	2,846	(97.7)	716	(95.7)	1,747	(98.4)	383	(98.2)		
Salary	< 2 million won	39	(1.3)	8	(1.1)	27	(1.5)	4	(1.0)	48.445	< 0.001
	2–3 million won	1,659	(57.0)	351	(46.9)	1,054	(59.4)	254	(65.1)		
	≥ 3 million won	1,215	(41.7)	389	(52.0)	694	(39.1)	132	(33.8)		
Workload	Very low	2	(0.1)	1	(0.1)	1	(0.1)	0	0.0	77.430	< 0.001
	Low	12	(0.4)	4	(0.5)	6	(0.3)	2	(0.5)		
	Moderate	935	(32.1)	294	(39.3)	467	(26.3)	174	(44.6)		
	High	1,603	(55.0)	377	(50.4)	1,051	(59.2)	175	(44.9)		
	Very high	361	(12.4)	72	(9.6)	250	(14.1)	39	(10.0)		
Nursing care delivery	Patient allocation	2,320	(79.6)	684	(91.4)	1,348	(75.9)	288	(73.8)	87.315	< 0.001
	Task allocation	593	(20.4)	64	(8.6)	427	(24.1)	102	(26.2)		
Practice guideline	Yes	2,383	(81.8)	619	(82.8)	1,441	(81.2)	323	(82.8)	1.184	0.553
	No	530	(18.2)	129	(17.2)	334	(18.8)	67	(17.2)		
Deviation from staffing standards	Yes	681	(23.4)	79	(10.6)	480	(27.0)	122	(31.3)	95.500	< 0.001
	No	2,232	(76.6)	669	(89.4)	1,295	(73.0)	268	(68.7)		

### Hospital characteristics

Nurses from 119 hospitals participated in this study. Characteristics of hospitals are presented in Table 2. This study included 18 tertiary hospitals, 67 general hospitals, and 34 semi-hospitals. Bed sizes of hospitals differed according to the hospital type, with tertiary hospitals having more than 500 beds, general hospitals having 100–999 beds, and semi-hospitals having less than 300 beds. For nurse staffing, RN staffing and NA staffing were separately reported as average nurse-to-patient ratios. While the coverage of RN staff varied by hospital type, the coverage of NA staff was similar among all types of hospitals. Since middle levels of RN staffing were 1:6, 1:10, and 1:12 in tertiary hospitals, general hospitals, and semi-hospitals, respectively, the proportion of hospitals with middle staffing levels was the highest in this study. Average HPPD by RN was 3.0 h in this study. HPPD was highest in tertiary hospitals (4.2 h), followed by that in general hospitals (2.7 h) and semi-hospitals (2.1 h). NA staffing had only three standards (i.e., 1:25, 1:30, and 1:40). RN staffing in tertiary hospitals was higher than those in other hospitals. Thus, NA staffing in tertiary hospitals was relatively low (0.7 HPPD). For other types of hospitals, most of them applied the middle level standard (1:30) for NA staffing (0.8 HPPD). The proportion of hospitals with inpatient systems for COVID-19 patients accounted for about 28% in tertiary hospitals and general hospitals, respectively. However, there were no inpatient care units for COVID-19 patients in semi-hospitals.

**Table 2:** Hospital characteristics

		Total (*n* = 119)		Tertiary hospital (*n* = 18)		General hospital (*n* = 67)		Semi-hospital (*n* = 34)	
Bed size	≥ 1000 beds	5	(4.2)	5	(27.8)				
	700–999 beds	13	(10.9)	9	(50.0)	4	(6.0)		
	500–699 beds	12	(10.1)	4	(22.2)	8	(11.9)		
	300–499 beds	21	(17.6)			21	(31.3)		
	200–299 beds	32	(26.9)			27	(40.3)	5	(14.7)
	100–199 beds	19	(16.0)			7	(10.5)	13	(38.2)
	< 100 beds	17	(14.2)					16	(47.1)
Nurse staffing (Nurse-to-patient ratio)									
RN	1:5	4	(3.4)	4	(22.2)				
	1:6	11	(9.2)	11	(61.1)				
	1:7	2	(1.7)	1	(5.6)	1	(1.5)		
	1:8	22	(18.5)			22	(32.8)		
	1:10	45	(37.8)			40	(59.7)	5	(14.7)
	1:12	32	(26.9)			4	(6.0)	28	(82.4)
	1:14	1	(0.8)					1	(2.9)
	HPPD	3.0 ± 0.8		4.2 ± 0.4		2.7 ± 0.3		2.1 ± 0.2	
NA	1:25	27	(22.7)			18	(26.9)	9	(26.5)
	1:30	72	(60.5)	9	(50.0)	44	(65.7)	19	(55.9)
	1:40	19	(16.0)	9	(50.0)	4	(6.0)	6	(17.6)
	HPPD	0.8 ± 0.1		0.7 ± 0.1		0.8 ± 0.1		0.8 ± 0.1	
COVID-19 care center	No	95	(79.8)	13	(72.2)	48	(71.6)	34	(100.0)
	Yes	24	(20.2)	5	(27.8)	19	(28.4)	0	(0.0)

### Nurses’ job satisfaction by personal, work environment, and hospital factors

Table 3 presents nurses’ job satisfaction by personal, work environment, and hospital factors. Among personal factors, nurses’ satisfaction differed according to nurses’ age and work experience in the current department. Nurses aged 40 and over had the highest job satisfaction, which was significantly higher than those in other age groups (F = 44.300, *p* < 0.001). The shorter the working experience in the current ward, the higher the job satisfaction (F = 3.290, *p* = 0.020). Among work environment factors, nurses’ satisfaction differed according to shift, salary, workload, nursing care delivery, and guideline. Nurses who had only day and evening shifts had the highest job satisfaction among all shift types of work (F = 14.430, *p* < 0.001). Nurses with a monthly salary of 3 million won or more had higher job satisfaction than other groups (F = 20.080, *p* < 0.001). The higher the workload, the lower the job satisfaction (F = 234.840, *p* < 0.001). Satisfaction of nurses who practiced patient allocation was significantly higher than that of nurses who practiced team nursing (t = 4.672, *p* < 0.001). Nurses who worked in hospitals with nursing practice guidelines had higher satisfaction than those in other groups (t = -7.741, *p* < 0.001).

**Table 3:** Differences of nurse’s satisfaction by personal, work environment, and hospital factors

		*n*	Mean	S.D.	t/F	*p*-value
**Personal factors**						
Gender	Male	120	5.76	1.73	1.767	0.077
	Female	2,792	5.47	1.78		
Age	< 30 years	1,856	5.29	1.76	44.300	< 0.001
	31–39 years	702	5.58	1.74		
	≥ 40 years	354	6.23	1.75		
Work experience in current department	< 1 year	898	5.60	1.78	3.290	0.020
	1–2 years	812	5.51	1.76		
	2–3 years	432	5.41	1.80		
	≥ 3 years	770	5.34	1.78		
**Work environment factors**						
Shift	Rotating three-shift	2,518	5.39	1.76	14.43	< 0.001
	Rotating two-shift	34	5.91	2.05		
	No-night rotating shift	228	6.30	1.64		
	Night-fixed shift	110	5.49	1.96		
	Fixed shift	22	5.77	2.02		
Employment type	Part-time	67	5.76	1.58	1.321	0.187
	Full-time	2,845	5.47	1.78		
Salary	< 2 million won	39	5.49	1.92	20.080	< 0.001
	2–3 million won	1,659	5.30	1.72		
	≥ 3 million won	1,214	5.72	1.83		
Workload	Low	12	6.67	1.44	234.840	< 0.001
	Moderate	935	6.46	1.49		
	High	1,602	5.22	1.60		
	Very high	363	4.02	1.84		
Nursing care delivery	Patient allocation	2,319	5.55	1.76	4.672	< 0.001
	Task allocation	593	5.17	1.83		
Guideline	No	530	4.94	1.77	-7.741	< 0.001
	Yes	2,382	5.60	1.76		
Over staffing standards	No	2,231	5.47	1.79	-0.490	0.624
	Yes	681	5.51	1.76		
**Hospital factors**						
Hospital type	Tertiary hospital	748	5.63	1.80	10.42	< 0.001
	General hospital	1,774	5.36	1.75		
	Semi-hospital	390	5.73	1.84		
Bed size	≥ 1000 beds	217	6.20	1.87	9.950	< 0.001
	700–999 beds	475	5.40	1.80		
	500–699 beds	530	5.43	1.63		
	300–499 beds	614	5.32	1.75		
	200–299 beds	535	5.32	1.80		
	100–199 beds	374	5.49	1.75		
	< 100 beds	167	5.96	1.90		
RN staffing	1:5	215	6.08	1.98	5.760	< 0.001
	1:6	518	5.43	1.69		
	1:7	30	5.17	1.72		
	1:8	886	5.32	1.73		
	1:10	906	5.48	1.78		
	1:12	352	5.57	1.82		
	1:14	5	6.20	1.79		
NA staffing	1:25	421	5.30	1.76	10.580	< 0.001
	1:30	2,086	5.44	1.75		
	1:40	405	5.83	1.91		
COVID-19 care	Yes	814	5.50	1.88	-0.428	0.669
	No	2,098	5.47	1.74		

Of hospital factors, nurses’ satisfaction differed according to hospital type (F = 10.420, *p* ≤ 0.001), bed sizes (F = 9.950, *p* < 0.001), RN staffing (F = 5.760, *p* < 0.001), and NA staffing (F = 10.580, *p* < 0.001). Nurses who worked in general hospitals had lower satisfaction than those in other groups. In addition, nurses who worked in big-sized hospitals having 1000 beds showed the highest satisfaction. Nurses’ satisfaction was more than 6 points when RN staffing was 1:5 or 1:14. In addition, nurses’ satisfaction level was the highest when hospitals had 1:40 NA staffing.

### Influencing factors for nurses’ satisfaction

Results of the mixed-effect model are shown in Table 4. This study identified factors from difference analysis and entered significant factors into the mixed-effect model. There was no multicollinearity because the variance inflation factor (VIF) was less than threshold values (mean: 1.93, maximum: 6.64). Age, shift, workload, and practice guidelines were significant factors influencing nurses’ satisfaction in this study. There was no significant factor among hospital factors. The satisfaction level of nurses who only worked day and evening shifts was higher than that of nurses who worked in three shifts (*p* = 0.001). In addition, the higher the workload, the lower the satisfaction (*p* < 0.001). Nurses’ satisfaction was found to be high when nursing units had practical guidelines (*p* < 0.001).

**Table 4:** Mixed-effects model of nurses’ satisfaction based on personal, work environment, and hospital factors

	Coeff.	S. E.	z	*p*-value	95% C.I.		
					Lower		Upper
**Personal factors**							
Age (base: < 30 years)							
31–39 years	0.26	0.07	3.610	< 0.001	0.12	0.40	
≥ 40 years	0.62	0.10	6.120	< 0.001	0.42	0.82	
Work experience in current department (Base: < 1 year)							
1–2 years	0.00	0.08	-0.020	0.982	-0.15	0.15	
2–3 years	-0.08	0.09	-0.900	0.370	-0.26	0.10	
≥ 3 years	-0.11	0.08	-1.340	0.181	-0.26	0.05	
**Work environment factors**							
Shift (Base: rotating three-shift)							
Rotating two-shift	0.38	0.27	1.440	0.151	-0.14	0.91	
No-night rotating shift	0.42	0.12	3.430	0.001	0.18	0.66	
Night-fixed shift	-0.04	0.15	-0.280	0.777	-0.35	0.26	
Fixed shift	0.46	0.34	1.370	0.169	-0.20	1.12	
Workload	-1.07	0.05	-22.65	< 0.001	-1.16	-0.97	
Delivery system (Base: patient allocation)	-0.01	0.08	-0.15	0.880	-0.17	0.15	
Employment type (Base: part-time)	-0.13	0.21	-0.630	0.527	-0.53	0.27	
Practice Guideline	0.43	0.07	5.750	< 0.001	0.28	0.58	
**Hospital factors**							
Hospital type (Base: tertiary hospital)							
General hospital	-0.07	0.27	-0.280	0.779	-0.60	0.45	
Semi-hospital	-0.02	0.39	-0.040	0.965	-0.79	0.75	
Bed size (Base: ≥ 1000 beds)							
700–999 beds	-0.37	0.27	-1.380	0.166	-0.91	0.16	
500–699 beds	-0.34	0.29	-1.180	0.238	-0.92	0.23	
300–499 beds	-0.26	0.33	-0.780	0.433	-0.91	0.39	
200–299 beds	-0.40	0.35	-1.140	0.256	-1.09	0.29	
100–199 beds	-0.41	0.37	-1.110	0.267	-1.14	0.31	
< 100 beds	-0.28	0.40	-0.700	0.485	-1.07	0.51	
HPPD by RN and NA	0.00	0.10	0.010	0.994	-0.20	0.20	
COVID-19 care	0.11	0.14	0.78	0.440	-0.17	0.38	
Constant	9.49	0.59	16.240	< 0.001	8.35	10.64	
Random effect (variance)							
Variance (95% C.I.)	2.29 (2.18–2.42)						
-log likelihood	-5383.26						
Wald chi(p)	738.67 (< 0.001)						

## Discussion

Average job satisfaction of nurses who worked in integrated care units was less than 6 points out of 10 points. Although it was difficult to make an absolute comparison due to different measurement tools and methods, the proportion of satisfied nurses was relatively low compared to those in other studies [3, 16]. Such low job satisfaction of nurses in this study might be attributed to the fact that this study was conducted during COVID-19 pandemic. During COVID-19 pandemic, frontline nurses caring for suspected or confirmed COVID-19 patients showed higher levels of exhaustion, stress, and burnout, which led to lower satisfaction and higher turnover [17, 18]. Even if they did not work at frontline, most nurses working at hospital experienced severe burnout, stress, and low satisfaction during the COVID-19 pandemic [19, 20]. Thus, although COVID-19 patients were not admitted to integrated care units, frequent staff rotations and high risk of infection due to COVID-19 might have contributed to nurses’ dissatisfaction.

Nurses’ satisfaction in this study was affected by several work environment factors rather than personal factors, consistent with other studies [1, 21]. Better work environment has been revealed to be a critical factor for better nurse outcomes such as job satisfaction, burnout, and turnover [1, 21]. Notably, work shift in this study was a significant factor affecting nurses’ satisfaction, with the highest level of satisfaction found for those with no-night rotating shift (i.e., rotating only day shift and evening shift) but the lowest level of satisfaction found for those with rotating three-shift and fixed night shift. Both rotating three-shift and fixed night shift included night shift, which could decrease sleep quality [12, 22] and diet quality [23]. Thus, nurses who had a night shift might be dissatisfied with their work due to poor sleep quality and diet quality as mentioned above. Due to the impact of rotating shift or night shift on psychosocial and physical health, the Korea government has included a strategy of night fixed-shift nurse in “Integrated Nursing and Care Service” scheme to reduce the burden of night shift work for the rotating shift nurse [13, 14]. Since no-night rotating nurses showed the highest satisfaction in this study, recruitment strategy for night fixed-shift nurse is evaluated to be effective. However, low percentage of no-night rotating shift nurses and low satisfaction of night fixed-shift nurses are still challenging.

Perceived workload is also a significant factor influencing nurses’ satisfaction. Although this study included several factors contributing to workload such as nurse staffing and shift, workload could not accurately be measured because nursing demands such as patient severity and patient ADL dependency by nursing unit were not considered in this study. Thus, the perceived workload was included to investigate the influence of workload on nurses’ satisfaction. Higher perceived workload was associated with lower job satisfaction of nurses, consistent with other previous studies [8, 24, 25]. Since nurse workload is related to patient-nurse ratio [26], integrated care unit could select adequate patient-nurse ratio by considering nursing demands such as patient severity and ADL dependency [14]. However, even if patient-nurse ratio is fixed, nursing demands change frequently according to characteristic of inpatients. Thus, high perceived workload might be an evidence of an inadequate nurse staffing. Since high workload affects not only nurse outcomes [8, 24], but also patient safety [27, 28], it is necessary to manage nurses’ workload by considering both nursing demands and nurse staffing.

Practice guideline affected nurses’ satisfaction in this study. Practice guideline refers to job description for roles of RN, NA, and other staff. Patients in integrated care units are provided with care services from a nursing team, including RN, NA, and other staff. RN leader of nursing team could delegate some services to NA or other staff according to practice guideline in integrated care units [14]. Effective delegation can improve nurses’ job satisfaction [29, 30] and patient outcomes [29, 31], allowing RNs to use more advanced nursing skills and facilitate more frequent observation of patients [32]. However, due to no clear instructions or documents describing delegation, many RNs hesitate to delegate to other nursing staff such as LPN or NA, considering it to be time consuming [33]. In addition, new graduate RNs are unsure of other nursing staffs’ roles and what tasks can be delegated [34]. Thus, effective delegation based on the practice guideline of an integrated care unit contributed to the increase of nurses’ job satisfaction.

In this study, factors affecting nurses’ job satisfaction were related to their work environment except for an individual’s age. In addition, most affecting factors could be modifiable. Thus, hospital and nurse managers should regularly monitor whether the current patient-nurse ratio is adequate for the unit and find ways to alleviate the burden of night shift. Moreover, they should utilize the practice guideline actively to ensure clear delegation in working as a team.

## Conclusion

During the COVID-19 pandemic, most nurses working at hospitals are not so satisfied with their work regardless of whether they work at frontline for patient care with COVID-19 or not. Nurses’ satisfaction is affected by several factors related to their work environment, such as shift work, workload perception, and delegation criteria. Low nurse satisfaction can have a substantial impact on both patient and nurse outcomes. It can be improved through organizational support. Therefore, nurse managers and organizations should actively investigate factors influencing satisfaction and develop strategies to improve satisfaction. To reduce nurses’ workload, hospital and nurse managers should evaluate the adequacy of nurse staffing and modify patient-nurse ratio according to nursing demands. In addition, the burden of night shift should be reduced by developing different strategies including recruitment strategy for night fixed-shift nurses. In integrated care system that provides nursing care as a team, nurse managers should encourage to use clear guidelines when delegating tasks among team members so that RN can more efficiently perform advanced level tasks focusing on patient safety.

## Data Availability

The datasets used and/or analyzed during the current study are available from the corresponding author on reasonable request.
